# A systematic review of theories, models and frameworks used to guide or evaluate the implementation of coercion reduction programs

**DOI:** 10.1192/j.eurpsy.2025.119

**Published:** 2025-08-26

**Authors:** T. Lantta, COST Action FOSTREN (Fostering and Strengthening Approaches to Reducing Coercion in European Mental Health Services)

**Affiliations:** 1 University of Turku, Turku, Finland; 2 Swinburne University of Technology, Melbourne, Australia; 3 University of Modena and Reggio Emilia, Reggio Emilia, Italy

## Abstract

**Abstract:**

Coercive practices in mental health care can infringe upon human rights, necessitating urgent global action to eliminate them. However, inconsistencies in clinical practice, fragmented research on effectiveness, and limited understanding of barriers and facilitators hinder the real-world transformation of services. As part of the COST Action FOSTREN (Fostering and Strengthening Approaches to Reducing Coercion in European Mental Health Services), Working Group 4 Implementation Science conducted a systematic review to examine the models, theories and frameworks employed by studies in implementing programs aimed at reducing coercion in mental health settings and the reported implementation outcomes. A comprehensive search was conducted across multiple databases, resulting in the inclusion of eight studies (nine papers). The identified coercion reduction programs utilized holistic approaches, risk assessment methods, staff training, and sensory modulation interventions. All of them were conducted in inpatient settings. Eight different implementation tools were identified, but none of the studies reported all sought implementation outcomes. The most frequently reported outcomes were acceptability and adaptation, while no studies provided data on implementation costs. Overall, the quality of the studies assessed was relatively low. The review highlights the underutilization of systematic implementation models, theories and frameworks when embedding coercion reduction interventions in routine mental health care, especially in emergency psychiatry settings. Further research, incorporating the perspectives of service users and carers, is needed to address this gap and determine the costs and resources required for implementing complex interventions with implementation models, theories and frameworks guidance.

**Disclosure of Interest:**

None Declared

